# Dose–Response Relationships Between Physical Activity, Dietary Behaviors, and Excess Body Weight: Identification of Behavioral Risk Patterns

**DOI:** 10.3390/nu18132104

**Published:** 2026-06-28

**Authors:** Jarosław Domaradzki

**Affiliations:** Department of Biological Principles of Physical Activity, Wroclaw University of Health and Sport Sciences, 51-612 Wroclaw, Poland; jaroslaw.domaradzki@awf.wroc.pl

**Keywords:** fruit and vegetable intake, fast food consumption, sweets consumption, behavioral risk patterns, exceed weight risk mapping

## Abstract

Background/Objectives: Dietary behaviors and physical activity are major risk factors of excess body weight; however, less is known about behavioral risk patterns and the role of physical activity in the context of unhealthy dietary patterns. The aim of this study was to identify behavioral risk patterns and multidimensional behavioral profiles associated with overweight/obesity and excess adiposity in physically active young adults. Methods: The cross-sectional study included 418 university students (199 men and 219 women). Physical activity was assessed using the IPAQ-LF, whereas dietary behaviors were evaluated using the QEB questionnaire. Healthy (fruit–vegetable) and unhealthy (fast food–sweets) dietary indices were derived from questionnaire responses. Logistic regression, predicted probability profiling, behavioral risk mapping, response surface analyses, and decision tree models were applied to evaluate behavioral associations with BMI- and FMI-defined outcomes. Results: The unhealthy dietary index emerged as the strongest behavioral predictor of excess body weight and adiposity. Higher fast food and sweets consumption was associated with increased odds of overweight/obesity (OR = 1.70, 95% CI: 1.31–2.24) and excess fat accumulation (OR = 1.96, 95% CI: 1.36–2.83). Physical activity demonstrated a positive association with BMI-defined overweight/obesity risk (OR = 0.69, 95% CI: 0.52–0.92), although no significant interaction effects were observed between physical activity and dietary behaviors. Multidimensional analyses identified distinct behavioral risk zones, whereas decision tree models indicated that unhealthy dietary behaviors represented the dominant factor underlying obesity-related risk classification. Conclusions: Unhealthy dietary behaviors demonstrated stronger associations with excess body weight and adiposity than protective dietary behaviors related to fruit and vegetable intake. Although physical activity was generally associated with lower obesity-related risk, unhealthy dietary behaviors showed stronger associations with adverse body composition outcomes.

## 1. Introduction

Children and adolescents with overweight or obesity are at increased risk of numerous adverse health outcomes [1]. Importantly, obesity occurring during childhood and adolescence markedly increases the likelihood of persistent obesity and cardiometabolic disorders in adulthood [1,2].

Because obesity frequently persists into adulthood, young adulthood represents a particularly important period for the development and maintenance of lifestyle behaviors associated with body weight regulation. Recent studies have demonstrated that dietary quality and physical activity jointly contribute to adiposity-related outcomes in adult populations, although their relative contributions and potential interactions remain incompletely understood [3,4,5]. Concurrent changes in diet quality and physical activity have been associated with changes in adiposity [3], while longitudinal evidence suggests complex relationships between physical activity, dietary behaviors, and body weight trajectories over time [4]. Furthermore, recent evidence indicates that combined dietary and physical activity behaviors may influence a broad spectrum of obesity-related health outcomes [5].

Excess body weight is a multifactorial condition influenced by behavioral, environmental, and lifestyle-related factors. Dietary behaviours and long-term dietary patterns are considered major determinants of body weight regulation and obesity risk. Contemporary evidence suggests that excess body weight is influenced not only by total caloric intake, but also by the qualitative and behavioural characteristics of food consumption patterns [6,7,8]. In recent decades, increasing attention has been directed toward the role of obesogenic dietary behaviours characterized by high intake of energy-dense, highly processed foods rich in fat and sugar, as well as insufficient consumption of nutrient-dense foods such as fruits and vegetables [9,10].

Previous studies have shown that healthier dietary patterns characterized by higher consumption of fruits, vegetables, whole grains, and minimally processed foods are associated with lower overweight and obesity risk, whereas Westernized dietary patterns rich in fast food, sweets, and processed products increase this risk [10,11]. A meta-analysis demonstrated that adherence to prudent or healthy dietary patterns was associated with a significantly reduced risk of overweight and obesity (OR = 0.64), while Western or unhealthy dietary patterns increased obesity risk (OR = 1.65) [10]. These findings support the concept that cumulative dietary behaviors and the balance between protective and obesogenic eating patterns play a central role in body weight regulation [11].

Young adulthood, particularly the transition to university life, represents a critical period for the development of long-term dietary habits [12,13]. Increased autonomy, changes in daily routines, irregular schedules, and greater exposure to convenience foods may contribute to the adoption of unhealthy eating behaviours, including increased consumption of sweets and fast food and insufficient intake of fruit and vegetables [12]. Importantly, healthy and unhealthy dietary behaviours frequently coexist rather than occur independently, resulting in complex behavioural patterns that may differentially influence excess body weight risk [13,14].

Longitudinal evidence further supports the importance of cumulative eating behaviors in obesity development [15]. Dietary patterns rich in fruits, vegetables, whole grains, and reduced-fat dairy products have been associated with smaller increases in BMI and waist circumference over time, whereas patterns characterized by sweets, processed foods, and fast food were linked to greater anthropometric deterioration [15]. More recent studies additionally highlight the contrasting metabolic effects of healthy and unhealthy dietary patterns. A “vegetable–fruit” dietary pattern has been associated with lower insulin resistance, reduced visceral fat accumulation, and lower risk of diabetes and central obesity, whereas a “sweets–fast food” pattern demonstrated positive associations with visceral adiposity and metabolic disturbances [16]. Collectively, these findings support the concept that the balance between protective dietary behaviors (e.g., fruit and vegetable intake) and obesogenic dietary behaviors (e.g., sweets and fast food consumption) may play a central role in body weight regulation and metabolic health.

Furthermore, previous analyses conducted within our broader research project demonstrated that fruit and vegetable intake, as well as the consumption of fast food and sweets, showed the strongest associations with BMI- and fat-related indicators among physically active young adults [17]. Therefore, fruit–vegetable and sweets–fast food indicators were selected as the principal dietary behavioral components in the present study.

Physical activity (PA) is widely recognized as one of the key behavioral determinants of body weight regulation and obesity prevention [18,19]. Through increased energy expenditure, improved metabolic flexibility, enhanced substrate utilization, and appetite regulation, regular physical activity contributes to the maintenance of energy balance and reduction in excess body weight risk [18]. However, the relationship between physical activity and obesity-related outcomes may be affected by behavioral and physiological responses that vary across individuals. Some studies suggest that exercise-induced energy deficits may be accompanied by increased caloric intake or reductions in non-exercise activity [20], whereas others report that appropriately performed exercise interventions generally increase total daily energy expenditure without substantial compensatory responses [21]. Together, these findings indicate considerable inter-individual variability in responses associated with physical activity.

Importantly, the relationship between physical activity and body weight regulation may depend strongly on the surrounding dietary environment [18]. Energy-dense diets combined with low levels of physical activity may disrupt appetite regulation and contribute to positive energy balance [18,19]. Conversely, healthier dietary patterns may enhance the beneficial metabolic effects of physical activity and facilitate long-term weight maintenance. Consequently, higher physical activity levels were associated with lower obesity-related risk across a range of dietary behavior profiles [18,19], although the nature and strength of these associations remain insufficiently understood.

Despite the growing body of evidence linking dietary behaviors and physical activity with excess body weight, several important limitations remain. Most previous studies have focused primarily on linear associations, simple correlations, or isolated behavioral factors. Consequently, substantially less is known about multidimensional behavioral patterns, interaction effects, and nonlinear relationships between healthy eating, unhealthy eating, and physical activity. Moreover, the interplay between physical activity and obesogenic dietary behaviors remains insufficiently understood.

To the best of our knowledge, no previous studies have simultaneously examined multidimensional behavioral risk patterns for healthy and unhealthy dietary behaviors across different physical activity levels. The novelty of the present study lies not only in examining the joint associations between dietary behaviors, physical activity, and overweight/obesity risk, but also in attempting to reduce complex lifestyle assessment into a parsimonious set of practically meaningful indicators. Instead of analyzing numerous isolated dietary variables, the study focused on simplified healthy (fruit–vegetable) and unhealthy (fast food–sweets) behavioral indices and evaluated their predictive utility in relation to physical activity patterns. This approach addresses an important methodological and practical gap, namely the need for simplified yet informative behavioral models that may improve screening feasibility, interpretability, and translation into public health recommendations.

Therefore, the aim of this study was to identify multidimensional behavioral risk patterns and classification profiles for healthy dietary behaviors, unhealthy dietary behaviors, and physical activity associated with overweight and obesity risk while accounting for their interactive effects. Specifically, the study aimed to answer the following questions: (1) Are healthy and unhealthy dietary behaviors independently associated with overweight/obesity risk? (2) Does physical activity modify the relationship between dietary behaviors and excess body weight risk? (3) Can specific combinations of physical activity and dietary behaviors define low-risk and high-risk behavioral zones? (4) Can decision tree models generate practical behavioral rules for overweight/obesity risk classification?

## 2. Materials and Methods

This study is part of a broader research series investigating the relationships between dietary behaviors, physical activity, and health-related outcomes among young physically active adults. In the present analysis, data from two independent student samples collected between 2022 and 2023 were combined into a single analytical dataset including only participants with complete information on dietary habits and physical activity.

### 2.1. Study Design

The present cross-sectional study was conducted among students of the Wroclaw University of Health and Sport Sciences during the 2022–2023 academic period. Two independent cohorts examined under identical study procedures and standardized measurement conditions were combined into a single analytical dataset. The assessment protocol included anthropometric measurements and body composition together with validated questionnaires evaluating dietary behaviours (QEB) and physical activity (IPAQ-LF).

### 2.2. Ethics

The study protocol was approved by the Senate Research Ethics Committee of the Wroclaw University of Health and Sport Sciences (approval no. 13/2022). Prior to participation, all individuals received detailed information regarding the study aims and procedures and provided informed electronic consent in accordance with the principles of the Declaration of Helsinki.

### 2.3. Sample Size

The required sample size was estimated using a standard formula for proportion-based studies at a 95% confidence level:

$$n=(\frac{1.96}{\delta }{)}^{2}\times p\left(1-p\right)$$

where *δ* represents the margin of error and *p* the expected proportion. Assuming the most conservative scenario (*p* = 0.5) and a margin of error of 0.05, the minimum required sample size was estimated at 384 participants. After accounting for a potential 20% reduction due to incomplete data, the target sample size was increased to approximately 460 participants.

The final analytical sample included 418 students with complete data on the variables relevant to the present analyses. This sample size was considered sufficient for the planned multivariable models and interaction analyses.

### 2.4. Participants

**Figure 1:**
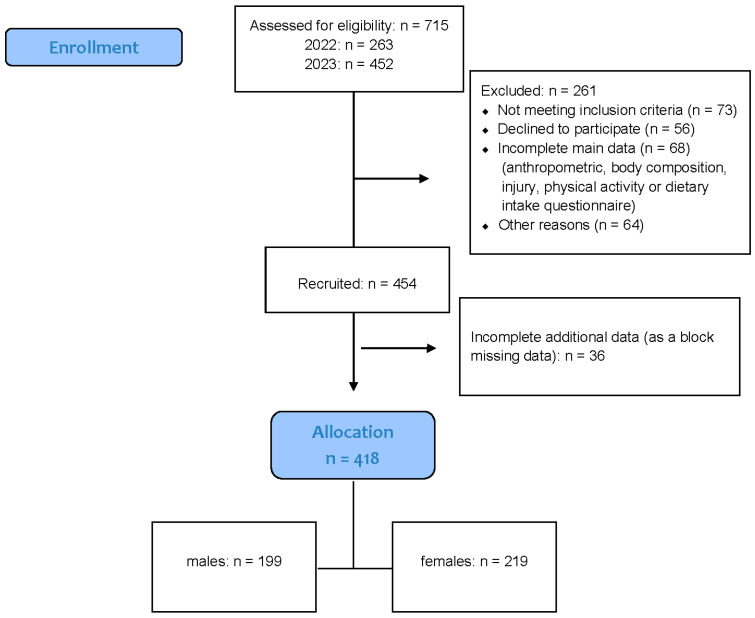
Flow diagram of the progress through all phases of data collection.

A total of 454 university students were recruited across two independent cohorts examined between 2022 and 2023. After applying the predefined inclusion and exclusion criteria, as well as data quality procedures, the final analytical sample comprised 418 participants (199 men and 219 women) with complete data on all variables included in the present analyses. Cases with isolated missing values were retained using the imputation procedure described in Section 2.8, whereas participants with substantial missing data were excluded from further analyses. A detailed participant flow diagram is presented in Figure 1.

Eligible participants were physically active full-time students aged 18–25 years who regularly attended in-person university classes. Individuals involved in university-regulated elite sport programmes were excluded to maintain a relatively homogeneous population of recreationally active young adults. Additional exclusion criteria included chronic metabolic or psychiatric diseases, night-shift work, and implausible questionnaire responses.

To justify merging the two cohorts into a single analytical dataset, baseline comparability was assessed for age, sex distribution, BMI, dietary behaviours, and physical activity indicators. No significant between-cohort differences were observed (all *p* > 0.05), supporting cohort equivalence and pooled analyses. Although the broader database has been used in previous publications addressing different research questions, the present study addresses a distinct research question and employs a different analytical framework focused on dietary behaviours, physical activity, and adiposity-related outcomes.

### 2.5. Anthropometric Measurements

Anthropometric assessments were conducted at the Biokinetics Research Laboratory of the Central Research Laboratory at the Wroclaw University of Health and Sport Sciences under standardized measurement conditions. Body height was measured twice using a calibrated GPM anthropometer (GPM Instruments GmbH, Susten, Switzerland) with an accuracy of 0.1 cm. Body mass and fat mass were assessed using a bioelectrical impedance analyzer (InBody230; InBody Co., Seoul, Republic of Korea) and recorded to the nearest 0.1 kg.

To minimize potential variability in bioelectrical impedance measurements, participants were instructed to avoid consuming food and beverages prior to testing whenever possible. They were additionally asked to refrain from eating a large evening meal on the day preceding the assessment and to maintain an overnight fasting period of approximately 12 h before measurement (i.e., no later than 22:00 for morning assessments).

All measurements were performed during scheduled academic sessions according to standardized procedures and manufacturer recommendations to reduce variability associated with hydration status and recent exercise. Fat Mass Index (FMI) was calculated as fat mass (kg) divided by height squared (m^2^). Excess body weight was defined as BMI ≥ 25 kg/m^2^. FMI-defined excess adiposity was classified using sex-specific cut-off points of 9 kg/m^2^ for females and 6 kg/m^2^ for males [22].

### 2.6. Questionnaire Measurements

#### 2.6.1. Physical Activity—International Physical Activity Questionnaire (IPAQ)

Physical activity was assessed using the Polish version of the International Physical Activity Questionnaire—Long Form (IPAQ-LF) [23], administered electronically via Google Forms. Questionnaire responses were converted into metabolic equivalent task minutes per week (MET-min/week) according to standardized IPAQ scoring procedures, providing estimates of total physical activity and sedentary behaviour. In the present study, physical activity variables were analysed as behavioural indicators rather than measures of training load or athletic performance.

Although the IPAQ is a widely used and internationally validated instrument, self-reported physical activity measures may be affected by recall bias and social desirability bias. Consequently, the estimated physical activity levels should be interpreted as approximations of habitual activity patterns rather than precise measures of energy expenditure.

For selected analyses, participants were additionally classified into three physical activity categories (low, moderate, and high) according to the standard IPAQ scoring protocol. Individuals classified as “low” did not meet the criteria for moderate or high physical activity. The “moderate” category included participants accumulating at least 600 MET-min/week through combinations of walking, moderate-intensity, or vigorous-intensity activities. The “high” category included participants accumulating at least 1500 MET-min/week of vigorous physical activity or at least 3000 MET-min/week of total physical activity [23]. These categories were subsequently used in the risk-zone and decision tree analyses to facilitate interpretation of behavioral risk patterns.

#### 2.6.2. Dietary Intake Questionnaire—Questionnaire Eating Behaviours (QEB)

Dietary behaviours during the previous 12 months were evaluated using the Questionnaire of Eating Behaviours (QEB) [24]. The QEB served as the basis for the later KomPAN questionnaire developed by the Committee of Human Nutrition Science of the Polish Academy of Sciences. Validation studies of the KomPAN instrument demonstrated satisfactory reproducibility of dietary frequency items (Fleiss’ kappa: 0.64–0.84) [25]. The present study focused on selected dietary indicators derived from the validated QEB framework rather than detailed nutrient intake estimation. The recommended 16-item core version of the questionnaire was applied [24].

Participants reported the frequency of consumption of selected food products using a six-point response scale ranging from “never” to “several times per day”, which was subsequently converted into average daily intake frequencies according to the standardized QEB scoring procedure.

For the purposes of the present study, two composite dietary indicators were derived: a healthy dietary index based on fruit and vegetable consumption and an unhealthy dietary index based on sweets and fast food consumption [17]. The healthy dietary index was constructed by combining the standardized frequencies of fruit and vegetable consumption, whereas the unhealthy dietary index was constructed by combining the standardized frequencies of sweets and fast food consumption. Higher values of the healthy dietary index reflected more frequent consumption of fruits and vegetables, whereas higher values of the unhealthy dietary index reflected more frequent consumption of sweets and fast food.

These two composite indicators were subsequently used as the principal dietary variables in all regression, probability-profile, risk-mapping, response surface, and decision tree analyses.

Because QEB-derived dietary variables demonstrated non-normal and discretized distributions, Yeo–Johnson transformation was applied to improve distributional properties and facilitate nonlinear modelling procedures. However, for interpretative purposes, identified classification patterns were subsequently back-transformed and related to the original frequency categories of food consumption.

The questionnaire was used to characterize habitual dietary behaviours rather than estimate precise nutrient intake, and the resulting indicators should therefore be interpreted as behavioral frequency measures rather than quantitative dietary exposure estimates.

### 2.7. Missing Data Handling

Missing data were identified in a small number of cases. To evaluate the missing-data mechanism, a logistic regression-based Missing Completely At Random (MCAR) diagnostic was performed, indicating that missingness was not associated with observed study variables [26,27].

Missing values were limited and occurred primarily in variables that were not included in the present analyses. Participants with incomplete data for the primary analytical variables, including body composition, physical activity, and dietary behaviors, were excluded prior to dataset construction. Consequently, the final analytical dataset used in the present study contained complete observations for all variables included in the statistical analyses, and no additional imputation procedures were required.

### 2.8. Statistics

Because the study aimed not only to quantify associations but also to identify multidimensional behavioral risk patterns and classification structures, a complementary analytical framework was applied. Logistic regression models were used to quantify the associations between dietary behaviors, physical activity, and obesity-related outcomes. Predicted-probability analyses were subsequently performed to visualize how obesity-related risk changed across the range of behavioral exposures. Risk-mapping procedures were used to identify behavioral risk zones associated with different levels of predicted risk. Response surface analyses enabled visualization of the combined relationships between healthy dietary behaviors, unhealthy dietary behaviors, and physical activity. Finally, decision tree models were applied to derive simplified and clinically interpretable classification rules describing obesity-related risk patterns.

The statistical analysis followed a multi-stage framework designed to characterize obesity-related risk across multiple complementary perspectives.

Initially, all variables were summarized using descriptive statistics, including means, standard deviations, medians, and 95% confidence intervals. Distributional assumptions were evaluated using the Shapiro–Wilk test. Because several variables demonstrated non-normal distributions, robust and nonparametric-oriented analytical approaches were additionally considered where appropriate. Preliminary sex comparisons were performed using standardized variables after appropriate transformation procedures when necessary.

#### 2.8.1. Stage 1: Association and Interaction Analyses

The first analytical stage focused on examining the independent and interactive associations between healthy dietary behaviours (fruit and vegetable intake), unhealthy dietary behaviours (sweets and fast food consumption), physical activity, and excess body weight indicators (Section 3.2 and Section 3.3). Logistic regression models with interaction terms were constructed to evaluate whether physical activity modified the relationships between dietary behaviours and overweight/obesity risk. Odds ratios (ORs) with 95% confidence intervals were reported. In addition, predicted probability profiles derived from regression models were used to visualize behavioural risk trajectories across different levels of dietary behaviours and physical activity.

#### 2.8.2. Stage 2: Behavioural Risk Mapping and Threshold Modelling

The second analytical stage aimed to identify multidimensional behavioural risk zones and classification patterns (Section 3.4). Response surface analyses, heatmaps, and isoline-based visualizations were generated from predicted probabilities to identify combinations of dietary behaviours and physical activity associated with low-risk and high-risk behavioural regions. These procedures enabled the exploration of multidimensional combinations of dietary behaviours and physical activity associated with varying levels of overweight and obesity risk.

#### 2.8.3. Stage 3: Decision-Rule Analyses

In the final stage, decision tree models were applied to derive simplified and clinically interpretable behavioural classification rules associated with excess body weight risk (Section 3.5). Decision trees were fitted using predefined IPAQ physical activity categories (low, moderate, high) and standardized dietary indices to approximate previously identified BMI- and FMI-based risk zones. To facilitate practical interpretation, decision tree split points derived from the unhealthy dietary index were translated into approximate behavioral profiles based on the original frequency coding system. Split-point values were interpreted as combinations of fast food and sweets consumption frequencies corresponding to the observed split points.

All analyses were conducted in Statistica 14.0 (TIBCO Software Inc., Palo Alto, CA, USA) and RStudio (2025.09). Statistical significance was set at *p* < 0.05.

### 2.9. AI Transparency Statement

Generative artificial intelligence (AI) tools were used in accordance with COPE and MDPI transparency recommendations and were limited to preparatory, technical, and editorial support during manuscript development. AI-assisted platforms supported literature exploration, manuscript organization, language refinement, and clarification of methodological terminology, but did not contribute to the study design, data collection, statistical decision-making, or interpretation of results.

Specifically, ChatAcademia (v.1.0, 2025) and Elicit (v.2.0, 2025) were used to facilitate literature exploration and refinement of research questions, whereas SciSpace (2025) assisted in organizing and reviewing scientific literature. In selected technical instances, draft code fragments and syntax suggestions were generated using Julius AI (2025) and ChatGPT (OpenAI, San Francisco, CA, USA, GPT-4.1, 2025) to support troubleshooting and clarification of R programming procedures. All AI-assisted outputs, including code suggestions and language edits, were manually reviewed, independently verified, and modified where necessary prior to implementation.

The author assumes full responsibility for the integrity, accuracy, reproducibility, and final content of the manuscript.

## 3. Results

### 3.1. General Characteristics of the Study Sample

The general characteristics of the study sample are presented in Table 1. Significant sex differences were observed for most anthropometric and behavioral variables. Males demonstrated greater body height, body weight, BMI, and overall physical activity levels, whereas females were characterized by higher FMI values and more frequent consumption of fruits and vegetables (all *p* < 0.001). In contrast, males reported higher frequencies of fast food consumption, sweetened beverage intake, and alcohol consumption (all *p* < 0.001). No significant sex differences were observed for sweets consumption (*p* = 0.436). The majority of both males and females were classified in the moderate IPAQ physical activity category (67.3% and 74.4%, respectively), whereas the high physical activity category was the least prevalent in both groups (8.6% and 6.9%, respectively). No significant sex-related differences were observed in the distribution of IPAQ physical activity categories (χ^2^ = 2.56, *p* = 0.279). Overall, these findings indicate distinct sex-specific anthropometric and behavioral profiles within the analyzed cohort.

### 3.2. Logistic Regression Analysis of Excess Body Weight Risk

Because BMI may incompletely reflect adiposity in physically active individuals, supplementary analyses (Table S1) were additionally performed using FMI as a body composition-based indicator of fat accumulation.

Logistic regression analysis demonstrated that male sex and older age were independently associated with higher odds of excess body weight (both *p* ≤ 0.001) (Table 2). Higher physical activity levels were associated with lower overweight/obesity risk (OR = 0.69, 95% CI: 0.52–0.92, *p* = 0.012), whereas the unhealthy dietary index, representing greater fast food and sweets consumption, was significantly associated with increased risk (OR = 1.70, 95% CI: 1.31–2.24, *p* < 0.001). The healthy dietary index demonstrated a nonsignificant trend toward a protective association (*p* = 0.091). No statistically significant interaction effects were observed between physical activity and dietary indices (both *p* > 0.05).

Logistic regression analysis based on sex-specific FMI thresholds demonstrated that older age and the unhealthy dietary index were independently associated with higher odds of excess fat accumulation (Table 3). The association for the unhealthy dietary index was slightly stronger in the FMI-based model (OR = 1.96, 95% CI: 1.36–2.83, *p* < 0.001) than in the BMI-based model. In contrast, physical activity demonstrated only a nonsignificant protective tendency (*p* = 0.100), whereas the healthy dietary index was not associated with FMI-defined excess fat risk (*p* = 0.765). Similarly, no significant interaction effects were observed between physical activity and dietary indices.

Overall, both models identified the unhealthy dietary index as the strongest behavioral predictor of adverse body composition outcomes. The BMI-based model additionally demonstrated a significant association of physical activity, whereas this relationship was weaker and nonsignificant in the FMI-based model.

The FMI-based model demonstrated lower residual deviance and AIC values than the BMI-based model, suggesting a comparatively better fit to the corresponding outcome data.

### 3.3. Predicted Probability Profiles

**Figure 2:**
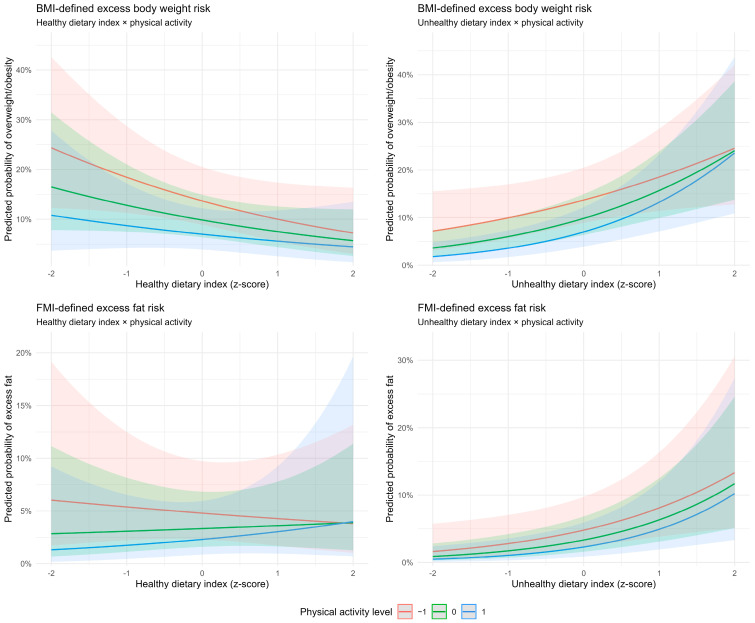
Predicted probability curves for BMI-defined excess body weight and FMI-defined excess fat according to healthy and unhealthy dietary indices at low (−1 SD), average (mean), and high (+1 SD) physical activity levels. Higher unhealthy dietary index values were associated with progressively greater predicted risk, whereas healthy dietary behaviors demonstrated weaker protective associations. Increasing physical activity shifted the probability curves downward without substantially altering their slopes, indicating predominantly independent relationship between physical activity and dietary behaviors. Colours indicate standardized physical activity levels: red = −1 SD, green = 0 SD, and blue = +1 SD. Shaded areas represent 95% confidence intervals.

Predicted probability analyses demonstrated consistent behavioral patterns across both BMI- and FMI-based models (Figure 2). Higher values of the unhealthy dietary index were associated with progressively greater predicted probability of overweight/obesity and excess fat accumulation across all physical activity levels. In contrast, increasing values of the healthy dietary index were associated with only modest reductions in predicted risk.

Across both models, higher physical activity levels demonstrated an overall downward shift in predicted risk profiles, indicating lower probability of adverse body composition outcomes at higher PA levels. However, the slopes of the predicted probability curves remained largely parallel between low, moderate, and high physical activity groups, consistent with the nonsignificant interaction effects observed in the logistic regression analyses. These findings suggest that physical activity exerted primarily an independent relationship rather than substantially modifying the relationships between dietary behaviors and excess body weight risk.

The FMI-based profiles additionally demonstrated steeper probability gradients for the unhealthy dietary index compared with the BMI-based model.

### 3.4. Behavioral Risk Mapping and Multidimensional Risk Profiling

**Figure 3:**
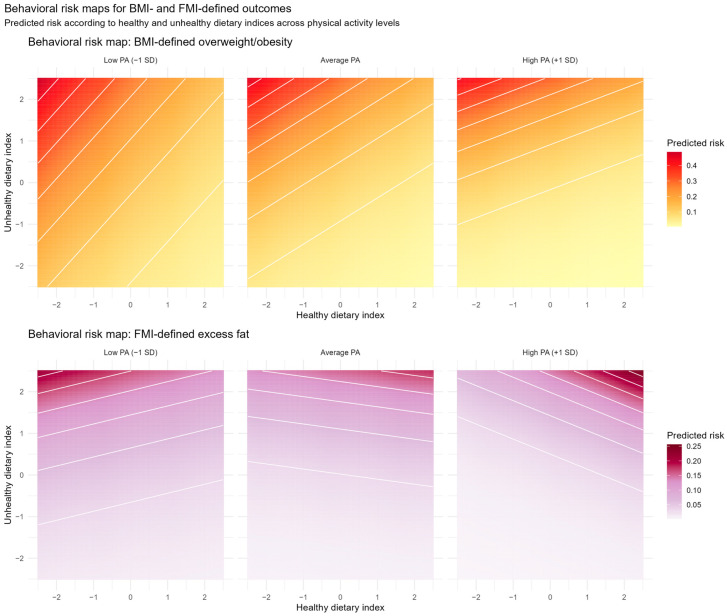
Behavioral risk maps for BMI- and FMI-defined outcomes. Heatmaps illustrating predicted risk of BMI-defined overweight/obesity ((**upper**) panels) and FMI-defined excess fat ((**lower**) panels) according to combinations of healthy and unhealthy dietary indices across low (−1 SD), average (mean), and high (+1 SD) physical activity levels. Warmer colors indicate higher predicted risk. White contour lines represent isolines of equal predicted probability and delineate multidimensional behavioral risk zones.

Behavioral risk maps based on predicted probabilities revealed clear multidimensional risk gradients across combinations of healthy and unhealthy dietary behaviors (Figure 3). In both BMI- and FMI-based models, the predicted probability of adverse body composition outcomes increased progressively with higher values of the unhealthy dietary index, whereas higher healthy dietary index values were generally associated with lower predicted risk.

The contour maps identified distinct behavioral risk zones. The highest predicted probabilities of overweight/obesity and excess fat accumulation were observed among individuals characterized by high unhealthy dietary index values combined with low healthy dietary index scores. In contrast, the lowest-risk zones were primarily associated with higher healthy dietary index values and lower unhealthy dietary behaviors.

Across all physical activity levels, higher PA shifted the overall risk distribution toward lower predicted probabilities; however, the overall configuration of the contour lines remained relatively stable. This pattern was consistent with the absence of statistically significant interaction effects observed in the logistic regression analyses and supports predominantly independent contributions of dietary behaviors and physical activity to excess body weight risk.

**Figure 4:**
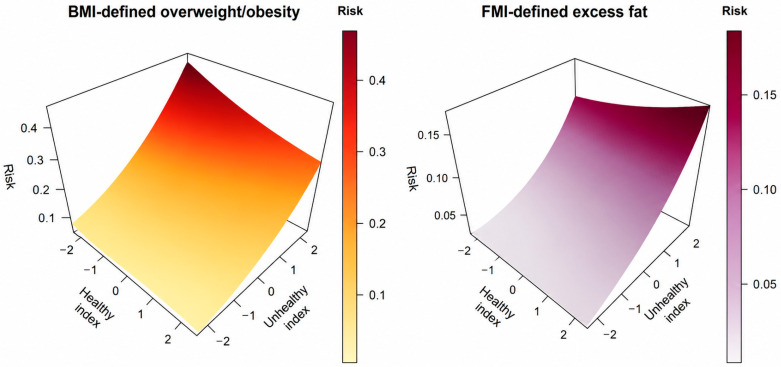
Response surface models for BMI- and FMI-defined outcomes. Three-dimensional response surface plots illustrating predicted probabilities of BMI-defined overweight/obesity (**left panel**) and FMI-defined excess fat (**right panel**) according to combinations of healthy and unhealthy dietary indices at average physical activity level. Surface elevation represents increasing predicted probability. The plots illustrate how predicted risk varies across combinations of healthy and unhealthy dietary behaviors.

Response surface analyses demonstrated clear multidimensional relationships between dietary behaviors and adverse body composition outcomes (Figure 4). In both BMI- and FMI-based models, predicted risk increased progressively across regions characterized by higher unhealthy dietary index values and lower healthy dietary index values.

Higher physical activity levels shifted the overall response surfaces toward lower predicted risk values; however, the overall surface geometry remained relatively stable, supporting the predominantly independent relationship observed in the logistic regression analyses.

Compared with the BMI-based model, FMI-derived response surfaces demonstrated sharper gradients and greater curvature within high-risk behavioral regions.

Probability-based risk mapping identified distinct behavioral risk zones across physical activity levels. In the BMI-based models, high-risk regions (predicted probability ≥ 30%) were primarily observed among individuals characterized by elevated unhealthy dietary index values combined with lower healthy dietary index scores. Importantly, higher physical activity levels shifted the distribution of high-risk regions toward greater unhealthy dietary index values, indicating lower predicted risk at comparable dietary exposure levels.

In the FMI-based models, elevated-risk zones were more concentrated and largely restricted to individuals simultaneously characterized by high unhealthy dietary index values and low-to-moderate healthy dietary index scores. Higher physical activity levels additionally reduced the extent of elevated FMI-related risk regions.

### 3.5. Decision Tree Classification Models

Complexity parameters indicated substantially greater structural complexity and higher cross-validation error for the BMI-based decision tree compared with the FMI-based model (Table 4).

The distribution of participants across behavioral risk zones differed considerably between BMI- and FMI-based models (Table 5). In the BMI-based model, participants were distributed across all four risk categories, with the majority classified as moderate risk (51.9%) or elevated risk (31.3%). In contrast, the FMI-based model was dominated by low-risk classifications (91.1%), whereas no participants were assigned to elevated- or high-risk categories.

**Figure 5:**
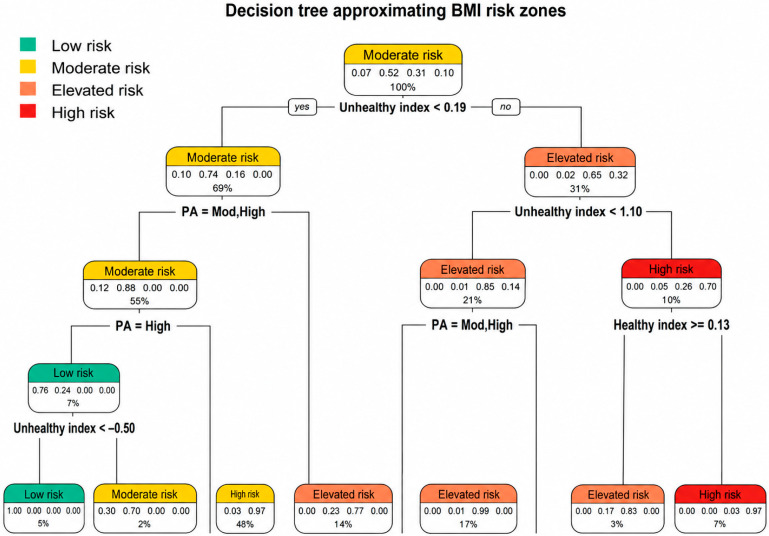
Decision tree illustrating BMI-defined behavioral risk zones. The tree illustrates hierarchical classification rules based on unhealthy dietary behaviors, healthy dietary behaviors, and physical activity categories. Values displayed within the nodes represent the probabilities of belonging to the low-, moderate-, elevated-, and high-risk categories, whereas percentages below each node indicate the proportion of participants assigned to the respective subgroup. Abbreviations: PA, physical activity category according to IPAQ classification; Mod, moderate physical activity.

Decision tree analysis demonstrated substantially greater classification complexity for the BMI-based risk-zone model compared with the FMI-based model. In the BMI model, all three predictors (physical activity category, healthy dietary index, and unhealthy dietary index) contributed to the classification process, resulting in an eight-terminal-node tree structure (Figure 5). The first and strongest partition was determined by the unhealthy dietary index (split point = 0.19), whereas subsequent splits additionally incorporated physical activity category and healthy dietary index.

**Figure 6:**
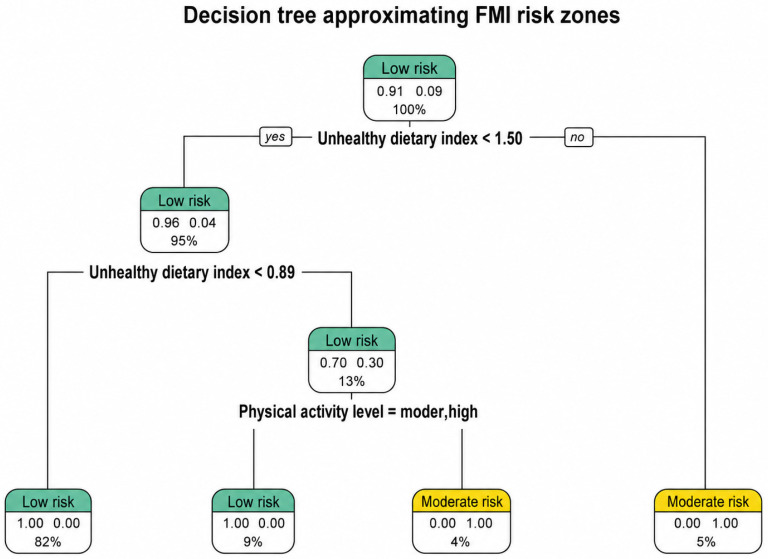
Decision tree illustrating FMI-defined behavioral risk zones for excess adiposity. The tree illustrates hierarchical classification rules primarily based on unhealthy dietary behaviors and physical activity levels. Values inside the nodes represent class probabilities for the identified risk zones, whereas percentages below the nodes indicate the proportion of participants classified within each node.

In contrast, the FMI-based model produced a markedly simpler tree structure (Figure 6). Only the physical activity category and the unhealthy dietary index contributed to the classification process, whereas the healthy dietary index was not retained in the final model. Furthermore, only low- and moderate-risk classifications were identified, resulting in substantially lower model complexity and cross-validation error compared with the BMI-based model.

The decision tree partitions corresponded to interpretable behavioral profiles based on the original dietary frequency coding. The lower-risk node (unhealthy dietary index < 0.89) approximately reflected patterns in which both fast food and sweets consumption remained at weekly or lower frequencies, whereas higher-risk nodes (>1.5) were primarily associated with combined frequent consumption of both unhealthy food categories (e.g., daily intake combined with several-times-per-week intake).

The BMI-based decision tree identified the unhealthy dietary index as the primary splitting variable defining BMI-related risk zones. Lower unhealthy dietary index values were associated with low-to-moderate risk profiles, particularly in participants with moderate or high physical activity levels. In contrast, higher unhealthy dietary index values shifted participants toward elevated and high-risk BMI categories. The tree structure additionally indicated lower predicted BMI-related risk among participants with moderate or high physical activity within lower and intermediate unhealthy dietary ranges. This pattern was less evident under more adverse dietary conditions. Furthermore, healthy dietary behaviors contributed to differentiating elevated and high-risk terminal nodes.

The FMI-based decision tree demonstrated a simpler and more diet-dominant structure. The unhealthy dietary index constituted the principal splitting variable, with lower values consistently associated with low FMI-related risk. Moderate FMI-related risk emerged primarily within intermediate-to-high ranges of the unhealthy dietary index. Physical activity differentiated risk only within the intermediate dietary range, whereas higher unhealthy dietary exposure was associated with moderate FMI risk regardless of physical activity level. Compared with the BMI tree, the FMI tree demonstrated a less behaviorally complex classification structure that relied primarily on unhealthy dietary behaviors.

## 4. Discussion

The present study demonstrated that obesogenic dietary behaviors, particularly frequent fast food and sweets consumption, were more strongly associated with excess body weight and adiposity than protective behaviors related to fruit and vegetable intake. Across all analytical approaches, the unhealthy dietary index consistently emerged as the strongest behavioral predictor of overweight/obesity and excess fat accumulation, whereas healthy dietary behaviors demonstrated comparatively weaker protective associations. Physical activity demonstrated a general protective association with lower predicted risk; however, no significant interaction effects were observed between physical activity and dietary behaviors. Although higher physical activity levels were generally associated with lower predicted obesity-related risk, unfavorable dietary behaviors remained consistently associated with higher-risk profiles. These findings suggest that physical activity and dietary behaviors were associated with obesity-related risk largely independently of one another. Importantly, the multidimensional analyses identified data-driven behavioral risk zones and simplified classification patterns. Decision tree models identified unhealthy dietary behaviors as the primary classification variable underlying obesity-related risk classification, whereas physical activity and healthy dietary behaviors contributed to further differentiation of risk categories. Additionally, BMI-based models demonstrated greater structural complexity, whereas FMI-based models showed steeper and more concentrated behavioral risk gradients.

### 4.1. Main Findings and General Interpretation

The present study demonstrated that unhealthy dietary behaviors, particularly frequent fast food and sweets consumption, were more strongly associated with excess body weight and adiposity than protective dietary behaviors related to fruit and vegetable intake. Across all analytical approaches, the unhealthy dietary index consistently showed the strongest associations with obesity-related risk, whereas physical activity demonstrated generally independent associations with obesity-related risk profiles. Previous studies have consistently demonstrated that dietary behaviors, physical activity, and sedentary behaviors are closely interrelated and jointly contribute to overweight and obesity risk [28,29]. Evidence from youth populations (including the Polish young adult population) indicates that dietary and physical activity patterns are interrelated and should be considered jointly when examining obesity-related risk [28,30]. Similarly, studies among adolescents have shown that recommended physical activity levels are associated with healthier dietary behaviors, whereas excessive screen time is linked to lower fruit and vegetable intake and higher fast food and sugar-sweetened beverage consumption [31].

Importantly, obesogenic dietary behaviors, particularly frequent fast food, sweets, and sugar-sweetened beverage consumption, may represent stronger predictors of excess body weight than isolated protective dietary components [29]. Collectively, these findings support the need for multidimensional behavioral approaches integrating dietary and physical activity patterns when examining overweight and obesity risk.

### 4.2. Unhealthy Dietary Behaviors as Dominant Predictors of Excess Body Weight

The findings suggest that obesogenic dietary behaviors may exert stronger effects on body composition than the isolated presence of healthy dietary components. Frequent consumption of fast food and sweets was consistently associated with higher predicted risk of overweight/obesity and excess adiposity across logistic, multidimensional, and classification-based analyses. The present findings are consistent with previous evidence showing that unhealthy dietary patterns characterized by frequent consumption of fast food, sweets, sugar-sweetened beverages, and processed foods are associated with a greater risk of overweight and obesity, whereas healthy dietary patterns rich in fruits, vegetables, and whole-grain products appear protective against excessive adiposity. Pachucki demonstrated that long-term unhealthful eating trajectories were prospectively associated with higher BMI values and an increased likelihood of overweight and obesity, emphasizing that dietary behaviors should be interpreted not only cross-sectionally but also within the context of behavioral trajectories over time [32]. Similarly, Newby et al. reported that dietary patterns based on high intake of fruits, vegetables, and whole grains were linked to smaller longitudinal increases in BMI and waist circumference, whereas patterns dominated by meat, processed foods, and refined products predicted greater adiposity gains [15].

The observed relationships are also supported by broader evidence derived from dietary pattern analyses. In a systematic review, Mu et al. showed that prudent/healthy dietary patterns were consistently associated with lower obesity risk, whereas Western/unhealthy patterns significantly increased the probability of overweight and obesity [10]. Comparable results were reported by Paradis et al., who found that individuals with high adherence to Western dietary patterns exhibited greater BMI, waist circumference, and fat mass, while prudent dietary patterns were associated with more favorable body composition profiles [33]. Moreover, Crovetto et al. demonstrated that low vegetable consumption and frequent intake of sugar-sweetened beverages constituted significant predictors of overweight and obesity among university students [34]. Together, these findings reinforce the interpretation that dietary quality operates not through isolated nutrients or single products, but rather through broader combinations of habitual eating behaviors.

However, the present findings should be interpreted cautiously because the dietary assessment did not provide information on total energy intake. Consequently, it was not possible to determine whether the observed associations were attributable primarily to specific dietary behaviors, broader dietary patterns, or differences in overall caloric consumption. In particular, the stronger associations observed for fast food and sweets consumption may partly reflect the higher energy density and caloric contribution of these foods rather than the unique effects of specific food categories. Therefore, the present results should be interpreted as associations with behavioral dietary patterns rather than evidence of specific causal dietary mechanisms.

At the same time, the present results should be interpreted with caution because eating behaviors represent highly complex and multidimensional constructs. Mesas et al., in a large systematic review, highlighted that evidence linking specific eating behaviors with obesity remains partly inconsistent due to methodological heterogeneity, variability in behavioral definitions, and difficulties in accurately capturing habitual dietary practices [35]. This limitation may partially explain why some dietary components in the present analyses demonstrated nonlinear associations and complex response patterns rather than simple linear trends. Importantly, the current findings extend previous research integrating dietary behaviors, physical activity, and obesity-related outcomes within a multidimensional framework. In our previous study, significant congruence between physical activity and dietary patterns was identified, including unhealthy behavioral triads composed of fast food, sweetened beverages, alcohol, sweets, and fried foods, alongside healthy fruit–vegetable combinations [17]. That study also showed that dietary behavior patterns were reflected in BMI, fat mass percentage, and FMI, particularly among females. The present results further support the notion that combinations of dietary behaviors and physical activity jointly contribute to obesity risk in a nonlinear and multidimensional manner.

### 4.3. Protective but Limited Role of Physical Activity

Although higher physical activity levels were associated with lower predicted risk of adverse body composition outcomes, no significant interaction effects were observed between physical activity and dietary behaviors. These findings suggest that physical activity and dietary behaviors were independently associated with obesity-related risk, with unhealthy dietary patterns showing stronger associations with adverse outcomes. The present findings also support the growing body of evidence indicating that dietary behaviors and physical activity should be analyzed jointly rather than as isolated lifestyle factors. Loprinzi et al. demonstrated that individuals who simultaneously maintained a healthy diet and sufficient physical activity presented the most favorable metabolic profiles, whereas an unhealthy diet combined with physical inactivity markedly increased the likelihood of metabolic syndrome [36]. Similarly, Koehler and Drenowatz emphasized that obesity prevention strategies focused on either diet or physical activity alone may be insufficient because these behaviors interact dynamically and may influence one another through complex behavioral and physiological pathways [37]. This interpretation is further supported by Shook, who highlighted the central role of physical activity in long-term energy balance regulation and body weight control [21]. Although some studies suggest that daily variability in lifestyle behaviors may operate relatively independently, physical activity variability itself appears protective for body composition outcomes [38]. Collectively, these findings reinforce the rationale for integrated multidimensional models examining combined lifestyle behaviors in relation to obesity risk.

### 4.4. Multidimensional Behavioral Risk Zones and Classification Patterns

Behavioral risk mapping and response surface analyses identified distinct multidimensional risk zones associated with combinations of healthy and unhealthy dietary behaviors. Importantly, higher physical activity levels shifted overall risk distributions toward lower probabilities, although the general configuration of behavioral risk gradients remained relatively stable. Despite the growing recognition that dietary behaviors and physical activity interact dynamically, multidimensional studies jointly examining these lifestyle domains remain relatively limited. Most previous investigations have focused on isolated behaviors, such as diet or physical activity alone, rather than on their combined behavioral patterns and interactions [37,38]. Existing evidence suggests that health-promoting and health-risk behaviors tend to cluster within individuals, forming distinct behavioral profiles characterized by combinations of dietary habits, physical inactivity, smoking, or alcohol consumption [39,40,41]. For example, Lippke et al. identified separate “health-promoting” and “health-risk” behavioral factors and demonstrated that nutrition and physical activity behaviors were more strongly interrelated than other lifestyle components [39]. Similarly, Schneider et al. revealed distinct clusters such as “Physically Inactives” and “Fruit and Vegetable Avoiders,” highlighting the coexistence of unhealthy dietary and activity patterns [40]. Comparable observations were reported among overweight women, in whom multiple co-occurring lifestyle risk behaviors were highly prevalent [41], while longitudinal population analyses confirmed the persistence of stable health behavior clusters over time [42,43].

However, although behavioral clustering approaches have become increasingly common, there is still a substantial lack of studies examining multidimensional risk zones, nonlinear risk gradients, and transition regions associated with combinations of dietary behaviors and physical activity. Consequently, the present study extends beyond traditional clustering or pattern identification approaches by exploring whether specific combinations of healthy and unhealthy dietary behaviors together with physical activity may generate heterogeneous obesity-related behavioral risk patterns.

### 4.5. Practical Utility of Decision Tree Models and Simplified Behavioral Indicators

Decision tree analyses demonstrated that simplified behavioral indices may provide interpretable classification patterns related to obesity risk. The identified risk-classification structures additionally suggest potential utility for screening, prevention strategies, and public health recommendations focused on reducing obesogenic dietary behaviors. Currently, studies based on decision tree approaches are becoming increasingly common because they enable multidimensional classification of individuals and identification of decision tree split points across multiple interacting variables. Such approaches are particularly valuable for detecting nonlinear relationships, subgroup-specific risk profiles, and interpretable behavioral patterns. Nevertheless, despite their growing popularity in medical and epidemiological research, decision tree analyses remain relatively uncommon in nutrition and lifestyle sciences, especially in studies simultaneously integrating dietary behaviors and physical activity patterns [44,45,46].

Previous studies have demonstrated the usefulness of decision tree models for obesity prediction and classification. Sarhan et al. reported high classification accuracy of obesity levels based on dietary and physical activity attributes among university students [44], whereas Iparraguirre-Villanueva et al. confirmed the effectiveness of decision tree models in predicting obesity status using behavioral and nutritional variables [45]. More recently, Trujillano et al. emphasized that classification trees are particularly useful for identifying informative split points, nonlinear interactions, and hidden subgroup-specific relationships in obesity-related risk stratification [46]. However, most previous applications focused primarily on predictive accuracy and obesity classification itself, rather than on identifying multidimensional behavioral risk zones and behavioral classification patterns integrating dietary and physical activity behaviors.

Therefore, the inclusion of decision tree-based analyses in the present study substantially enriches the methodological framework and helps address an important gap in the literature regarding multidimensional analyses of dietary behaviors and physical activity. In particular, the present approach allowed not only the classification of obesity-related profiles, but also the identification of multidimensional risk regions and combinations of healthy and unhealthy behaviors associated with increased obesity risk. The decision tree split points should be interpreted as model-derived risk profiles rather than dietary recommendations. Because the dietary indices were based on frequency-coded food items, the identified split points may be understood as approximate behavioral patterns, for example reflecting combinations of more frequent sweets and fast food consumption, rather than quantitative dietary intake estimates. In the BMI-based tree, unhealthy dietary patterns emerged as the primary discriminator of risk zones. Very low unhealthy dietary index values combined with high physical activity were associated with the lowest BMI-related risk, whereas increasing unhealthy dietary exposure progressively shifted participants toward elevated and high-risk profiles. Importantly, moderate-to-high physical activity was associated with more favorable BMI-related risk classifications within lower and intermediate unhealthy dietary ranges, whereas dietary patterns characterized by higher unhealthy dietary index values were more frequently associated with elevated-risk classifications. In contrast, the FMI-based tree demonstrated a simpler structure dominated by the unhealthy dietary index. Low FMI-related risk was primarily associated with lower unhealthy dietary exposure, whereas moderate FMI risk was primarily observed among participants characterized by intermediate and higher unhealthy dietary index values. Physical activity differentiated risk only within the intermediate dietary range, indicating that adiposity-related risk defined by FMI may be more strongly linked to cumulative unhealthy dietary behaviors than BMI-defined risk.

### 4.6. BMI Versus FMI as Behavioral Outcome Indicators

The BMI- and FMI-based models demonstrated partially different behavioral structures and risk gradients. Whereas BMI-based analyses showed greater classification complexity, FMI-based models demonstrated steeper and more concentrated behavioral risk gradients, suggesting potentially greater sensitivity for detecting adiposity-related behavioral patterns in physically active young adults. This finding should be interpreted in the context of the study population, which consisted of physically active young adults characterized by a relatively low prevalence of excess adiposity. Consequently, the FMI-based model primarily differentiated low- and moderate-risk profiles, with relatively few participants classified into higher-risk categories. Importantly, the rationale for using FMI was not to improve the analytical precision of the underlying bioelectrical impedance measurement itself. Rather, FMI provides a body-size-adjusted indicator of adiposity by standardizing fat mass relative to height squared. Consequently, FMI may offer a more biologically interpretable representation of excess adiposity than body fat percentage alone, particularly when comparing individuals with different body sizes and anthropometric characteristics. Similarly, fat mass expressed in kilograms represents absolute adiposity but does not account for differences in body size. Therefore, FMI was preferred as the primary adiposity indicator because it standardizes fat mass relative to height squared and facilitates comparisons across individuals with different anthropometric characteristics. The present findings may also be interpreted in the context of the growing evidence suggesting that fat mass index (FMI) constitutes a more sensitive indicator of adiposity and obesity-related health risk than BMI alone. Previous studies demonstrated that FMI may outperform BMI in identifying obesity-related metabolic disturbances and cardiovascular risk profiles. Salinas-Mandujano et al. reported that FMI showed strong discriminatory power for detecting cardiovascular risk factors among young adults and suggested that FMI and BMI should be jointly considered in obesity-related screening procedures [47]. Similarly, Pourshahidi et al. found that FMI was the strongest predictor of overall cardiometabolic risk compared with traditional anthropometric indicators [48]. Although BMI remains the most commonly used obesity indicator, it does not distinguish between fat mass and lean tissue, which may reduce its ability to accurately reflect metabolic risk [49,50]. Consequently, the clinical and epidemiological importance of FMI as a marker of excessive adiposity and cardiometabolic health has increased substantially in recent years. Liu et al. further demonstrated that FMI exhibited greater predictive utility for metabolic syndrome than both BMI and body fat percentage, while also allowing identification of clinically meaningful cut-off points associated with elevated metabolic risk [50]. Although some evidence indicates high correlations between BMI and FMI, BMI may underestimate the actual degree of adiposity because it cannot differentiate body composition components [48]. Therefore, the inclusion of FMI in the present analyses substantially strengthens the interpretation of obesity-related behavioral risk patterns. In particular, the observed nonlinear and multidimensional relationships between dietary behaviors, physical activity, and obesity outcomes may be more precisely captured when adiposity is assessed using FMI rather than BMI alone. Therefore, although the FMI-based model exhibited a narrower distribution of risk categories than the BMI-based model, it remained informative by identifying consistent behavioral gradients and stronger associations with unhealthy dietary behaviors.

The strengths of the study include the multidimensional analytical framework combining regression, probability profiling, risk mapping, response surface analyses, and decision tree modeling. However, several limitations should be acknowledged. First, the cross-sectional design precludes causal inference and limits the ability to determine temporal relationships between dietary behaviors, physical activity, and obesity-related outcomes. Consequently, the reported findings should be interpreted as associations rather than evidence of causal effects. Second, the behavioral variables were based on self-reported questionnaires, which may be affected by recall bias, social desirability bias, and potential misclassification of dietary intake or physical activity levels. In particular, self-reported physical activity assessed using the IPAQ may differ from objectively measured activity and may result in overestimation or underestimation of actual activity levels. Such measurement error could attenuate the strength of observed associations and contribute to uncertainty in the estimated relationships. In particular, dietary behaviors were assessed using a retrospective food-frequency questionnaire covering the previous 12 months. Although this approach is suitable for estimating habitual dietary patterns, it may be affected by recall bias, reporting inaccuracies, and exposure misclassification, potentially reducing the precision of estimated dietary behaviors. Third, the study sample was restricted to physically active university students aged 18–25 years, which may reduce the generalizability of the findings to less active populations, adolescents, older adults, or individuals with different socioeconomic and health characteristics. Although this relatively narrow age range improved sample homogeneity and reduced age-related variability in body composition, dietary behaviors, and physical activity patterns, the identified behavioral risk profiles may not fully reflect associations present in broader adult populations. Finally, a limitation is the lack of information on total energy intake. The dietary questionnaire assessed habitual food consumption frequency rather than quantitative nutrient or caloric intake. Consequently, it was not possible to determine whether the observed associations were attributable to specific dietary behaviors, overall dietary patterns, or differences in total energy consumption. Future studies incorporating detailed dietary assessment and energy intake estimation are needed to clarify the mechanisms underlying these associations.

The present findings suggest that preventive strategies should prioritize reduction in unhealthy dietary behaviors, particularly fast food and sweets consumption, even among physically active individuals. Future longitudinal and interventional studies are needed to determine whether the identified behavioral risk zones and classification patterns predict long-term obesity-related outcomes.

## 5. Conclusions

Frequent consumption of fast food and sweets was consistently associated with increased risk of excess body weight and excess fat accumulation, whereas healthy dietary behaviors showed comparatively weaker associations with obesity-related risk. Although physical activity was generally associated with lower obesity-related risk, unhealthy dietary behaviors demonstrated stronger and more consistent associations with adverse body composition outcomes.

Multidimensional analyses identified distinct behavioral risk zones and simplified classification patterns related to overweight/obesity and excess adiposity. Unhealthy dietary behaviors consistently showed the strongest associations with obesity-related risk across logistic regression, risk-mapping, and decision tree models, whereas physical activity was associated with risk profiles largely independently of dietary behaviors. The identified tree-based risk-classification patterns may provide a useful framework for behavioral obesity-risk profiling.

Additionally, FMI-based models demonstrated steeper and more concentrated behavioral risk gradients, whereas BMI-based models showed greater structural and behavioral complexity. These findings suggest that BMI and FMI may capture partially different dimensions of adiposity-related behavioral variability in physically active young adults.

## Figures and Tables

**Table 1:** Anthropometric characteristics, physical activity levels, and dietary behavior indicators in male and female participants. QEB-derived dietary variables were normalized using the Yeo–Johnson transformation.

	Sex = Males, N = 199				Sex = Females, N = 219					
Variables	Mean	95% CI		SD	Mean	95% CI		SD	t	*p*
		Lower	Upper			Lower	Upper			
Body height [cm]	182.2	181.2	183.2	7.1	168.2	167.4	169.0	6.0	21.85	<0.000
Body weight [kg]	79.6	78.3	81.0	9.9	60.9	59.7	62.1	9.0	20.29	<0.000
BMI [kg/m^2^]	24.0	23.6	24.3	2.5	21.5	21.1	21.9	2.7	9.74	<0.000
BFP [%]	16.2	15.5	16.8	4.62	23.2	22.5	24.0	5.65	−13.86	<0.000
BF [kg]	14.5	13.8	15.2	5.5	14.5	13.8	15.2	5.5	−3.02	0.003
FMI [kg/m^2^]	3.9	3.7	4.1	1.4	5.1	4.9	5.3	1.8	−7.33	<0.000
IPAQ [MET/min/week]	3608.2	3418.5	3797.9	1356.9	3019.8	2886.5	3153.1	1000.9	5.08	<0.000
Fruits [scores]	0.52	0.49	0.56	0.28	0.74	0.69	0.79	0.37	−6.60	<0.000
Vegetables [scores]	0.59	0.55	0.64	0.32	0.88	0.83	0.93	0.38	−8.24	<0.000
Fastfood [scores]	0.18	0.16	0.21	0.15	0.08	0.07	0.09	0.07	8.88	<0.000
Sweets [scores]	0.41	0.38	0.44	0.24	0.43	0.39	0.47	0.32	−0.78	0.436
IPAQ categories	Low: n = 48, (24.1%) Moderate: n = 134, (67.3%) High: n = 17, (8.6%)				Low: n = 41, (18.7%) Moderate: n = 163, (74.4%) High: n = 15, (6.9%)				χ^2^ = 2.56, *p* = 0.279	

**Table 2:** Logistic regression analysis of BMI-defined excess body weight risk (BMI ≥ 25 kg/m^2^).

Variable	B	SE	OR	95% CI for OR	*p*
Intercept	−17.798	3.798	—	—	<0.001
Male sex	0.989	0.309	2.69	1.48–4.99	0.001
Age	0.755	0.182	2.13	1.50–3.08	<0.001
Physical activity (PA_z)	−0.368	0.147	0.69	0.52–0.92	0.012
Healthy dietary index	−0.293	0.173	0.75	0.53–1.04	0.091
Unhealthy dietary index	0.532	0.137	1.70	1.31–2.24	<0.001
PA × healthy dietary index	0.061	0.143	1.06	0.80–1.42	0.668
PA × unhealthy dietary index	0.172	0.122	1.19	0.94–1.52	0.160
Model statistics: Residual deviance = 340.43; AIC = 358.43.					

**Table 3:** Logistic regression analysis of FMI-defined excess fat risk (females: FMI ≥ 9 kg/m^2^; males: FMI ≥ 6 kg/m^2^).

Variable	B	SE	OR	95% CI for OR	*p*
Intercept	−16.015	5.250	—	—	0.002
Male sex	0.582	0.485	1.79	0.70–4.80	0.230
Age	0.613	0.251	1.85	1.12–3.02	0.014
Physical activity (PA_z)	−0.381	0.231	0.68	0.44–1.09	0.100
Healthy dietary index	0.082	0.274	1.09	0.63–1.86	0.765
Unhealthy dietary index	0.674	0.185	1.96	1.36–2.83	<0.001
PA × healthy dietary index	0.203	0.223	1.22	0.80–1.92	0.362
PA × unhealthy dietary index	0.115	0.165	1.12	0.80–1.54	0.485
Model statistics: Residual deviance = 174.29; AIC = 190.29.					

**Table 4:** Structural complexity and cross-validation characteristics of decision tree models approximating BMI- and FMI-based behavioral risk zones.

Model	Variables Used	Final Tree Size	Minimum Cross-Validation Error
BMI risk zones	IPAQ category, healthy index, unhealthy index	8 terminal nodes	0.229
FMI risk zones	IPAQ category, unhealthy index	4 terminal nodes	0.027

**Table 5:** Distribution of participants across BMI- and FMI-based behavioral risk zones derived from probability-based risk classification.

Risk Zone	BMI Model n (%)	FMI Model n (%)
Low risk	28 (6.7%)	381 (91.1%)
Moderate risk	217 (51.9%)	37 (8.9%)
Elevated risk	131 (31.3%)	0 (0.0%)
High risk	42 (10.0%)	0 (0.0%)
Total	418 (100%)	418 (100%)

## Data Availability

The data presented in this study are available on request from the corresponding author. Access is restricted due to ethical and privacy considerations, as participants were informed during the consent procedure that individual-level data would not be publicly shared, and the combination of variables may allow potential participant re-identification.

## Supplementary Materials

The following supporting information can be downloaded at: https://www.mdpi.com/article/10.3390/nu18132104/s1, Table S1: Behavioral threshold ranges for BMI- and FMI-defined risk zones across physical activity levels.

## References

[1] World Health Organization (2015). Guideline: Sugars Intake for Adults and Children.

[2] Lobstein T., Baur L., Uauy R. (2004). Obesity in children and young people: A crisis in public health. Obes. Rev..

[3] Aryannezhad S., Imamura F., De Lucia Rolfe E., Griffin S.J., Wareham N.J., Brage S., Forouhi N.G. (2025). Concurrent Changes in Diet Quality and Physical Activity and Association with Adiposity in Adults. J. Netw. Open.

[4] Kim Y., Lee J.M., Kim J., Dhurandhar E., Soliman G., Wehbi N.K., Canedy J. (2017). Longitudinal Associations between Body Mass Index, Physical Activity, and Healthy Dietary Behaviors in Adults: A Parallel Latent Growth Curve Modeling Approach. PLoS ONE.

[5] Salem A., Ammar A., Trabelsi K., Boukhris O., Heydenreich J., Ghazzawi H.A., Amawi A.T., Grosso G., Zmijewski P., Jahrami H. (2026). Combined Diet and Physical Activity Effects on Health-Related Outcomes in People with Overweight or Obesity: An Overview of Systematic Reviews. Front. Nutr..

[6] Malhotra S., Sivasubramanian R., Singhal V. (2021). Adult obesity and its complications: A pediatric disease?. Curr. Opin. Endocrinol. Diabetes Obes..

[7] Almoziny A., Aldughaim A. (2016). Modifying Dietary Behaviors to Manage Body Weight. Int. J. Clin. Nutr..

[8] Hall K.D., Guo J. (2017). Obesity Energetics: Body Weight Regulation and the Effects of Diet Composition. Gastroenterology.

[9] Yao M., Roberts S.B. (2001). Dietary energy density and weight regulation. Nutr. Rev..

[10] Mu M., Xu L.F., Hu D., Wu J., Bai M.J. (2017). Dietary Patterns and Overweight/Obesity: A Review Article. Iran. J. Public Health.

[11] Koutras Y., Chrysostomou S., Poulimeneas D., Yannakoulia M. (2022). Examining the associations between a posteriori dietary patterns and obesity indexes: Systematic review of observational studies. Nutr. Health.

[12] Maillet M.A., Grouzet F.M.E. (2023). Understanding changes in eating behavior during the transition to university from a self-determination theory perspective: A systematic review. J. Am. Coll. Health.

[13] Julia C., Omorou A., Lecomte E., Langlois J., Touvier M., Hercberg S., Briançon S., Kesse-Guyot E., Guillemin F. (2023). Behavioural risk patterns in adolescents with excess weight participating in the PRALIMAP-INÈS trial. Public Health Nutr..

[14] Laska M.N., Hearst M.O., Lust K., Lytle L.A., Story M. (2015). How we eat what we eat: Identifying meal routines and practices most strongly associated with healthy and unhealthy dietary factors among young adults. Public Health Nutr..

[15] Newby P.K., Muller D., Hallfrisch J., Qiao N., Andres R., Tucker K.L. (2003). Dietary patterns and changes in body mass index and waist circumference in adults. Am. J. Clin. Nutr..

[16] Yin X., Chen Y., Lu W., Jin T., Li L. (2020). Association of dietary patterns with the newly diagnosed diabetes mellitus and central obesity: A community based cross-sectional study. Nutr. Diabetes.

[17] Domaradzki J. (2023). Congruence between Physical Activity Patterns and Dietary Patterns Inferred from Analysis of Sex Differences in Lifestyle Behaviors of Late Adolescents from Poland: Cophylogenetic Approach. Nutrients.

[18] Chaput J.P., Klingenberg L., Rosenkilde M., Gilbert J.A., Tremblay A., Sjödin A. (2011). Physical activity plays an important role in body weight regulation. J. Obes..

[19] Bourdier P., Simon C., Bessesen D.H., Blanc S., Bergouignan A. (2023). The role of physical activity in the regulation of body weight: The overlooked contribution of light physical activity and sedentary behaviors. Obes. Rev..

[20] Westerterp K.R. (2010). Physical activity, food intake, and body weight regulation: Insights from doubly labeled water studies. Nutr. Rev..

[21] Shook R.P. (2016). Obesity and energy balance: What is the role of physical activity?. Expert Rev. Endocrinol. Metab..

[22] Wong J.C., O’Neill S., Beck B.R., Forwood M.R., Khoo S.K. (2021). Comparison of obesity and metabolic syndrome prevalence using fat mass index, body mass index and percentage body fat. PLoS ONE.

[23] IPAQ Research Committee (2005). Guidelines for the Data Processing and Analysis of the International Physical Activity Questionnaire. https://sites.google.com/view/ipaq/score.

[24] (2025). Questionnaire of Eating Behaviour. https://docs.google.com/forms/d/e/1FAIpQLSdEKO_zdm_kGlom2Bq5Hxqun1lNJtyhcT2HHXBRj1dYah3P6w/viewform.

[25] Kowalkowska J., Wadolowska L., Czarnocinska J., Czlapka-Matyasik M., Galinski G., Jezewska-Zychowicz M., Bronkowska M., Dlugosz A., Loboda D., Wyka J. (2018). Reproducibility of a Questionnaire for Dietary Habits, Lifestyle and Nutrition Knowledge Assessment (KomPAN) in Polish Adolescents and Adults. Nutrients.

[26] Heymans M.W., Eekhout I. (2019). Applied Missing Data Analysis with SPSS and R (Studio). https://bookdown.org/mwheymans/bookmi/.

[27] Austin P.C., White I.R., Lee D.S., van Buuren S. (2021). Missing Data in Clinical Research: A Tutorial on Multiple Imputation. Can. J. Cardiol..

[28] Jezewska-Zychowicz M., Gębski J., Guzek D., Świątkowska M., Stangierska D., Plichta M., Wasilewska M. (2018). The Associations between Dietary Patterns and Sedentary Behaviors in Polish Adults. Nutrients.

[29] Barrington W.E., Beresford S.A.A. (2018). Applying Multiple Statistical Methods to Derive an Index of Dietary Behaviors Most Related to Obesity. Am. J. Epidemiol..

[30] Kelishadi R., Ardalan G., Gheiratmand R., Gouya M.M., Razaghi E.M., Delavari A., Majdzadeh R., Heshmat R., Motaghian M., Barekati H. (2007). Association of physical activity and dietary behaviours in relation to the body mass index in Iranian children and adolescents. Bull. World Health Organ..

[31] Lowry R., Michael S., Demissie Z., Kann L., Galuska D.A. (2015). Associations of Physical Activity and Sedentary Behaviors with Dietary Behaviors among US High School Students. J. Obes..

[32] Pachucki M.A. (2012). Food pattern analysis over time: Unhealthful eating trajectories predict obesity. Int. J. Obes..

[33] Paradis A.M., Godin G., Pérusse L., Vohl M.-C. (2009). Associations between dietary patterns and obesity phenotypes. Int. J. Obes..

[34] Crovetto M., Valladares M., Espinoza V., Mena F., Oñate G., Fernandez M., Durán-Agüero S. (2018). Effect of healthy and unhealthy habits on obesity: A multicentric study. Nutrition.

[35] Mesas A.E., Muñoz-Pareja M., López-García E., Rodríguez-Artalejo F. (2012). Selected eating behaviours and excess body weight: A systematic review. Obes. Rev..

[36] Loprinzi P.D., Smit E., Mahoney S. (2014). Physical activity and dietary behavior in US adults and their combined influence on health. Mayo Clin. Proc..

[37] Koehler K., Drenowatz C. (2022). Understanding the Interaction Between Physical Activity and Diet for the Promotion of Health and Fitness. Front. Nutr..

[38] Hooker S.A., Oswald L.B., Reid K.J., Baron K.G. (2020). Do Physical Activity, Caloric Intake, and Sleep Vary Together Day to Day? Exploration of intraindividual variability in 3 key health behaviors. J. Phys. Act. Health.

[39] Goldstein M.G., Whitlock E.P., DePue J. (2004). Multiple behavioral risk factor interventions in primary care. Am. J. Prev. Med..

[40] Lippke S., Nigg C.R., Maddock J.E. (2012). Health-promoting and health-risk behaviors: Theory-Driven Analyses of Multiple Health Behavior Change in Three International Samples. Int. J. Behav. Med..

[41] Sanchez A., Norman G.J., Sallis J.F., Calfas K.J., Rock C., Patrick K. (2008). Patterns and correlates of multiple risk behaviors in overweight women. Prev. Med..

[42] Schneider S., Huy C., Schuessler M., Diehl K., Schwarz S. (2009). Optimising lifestyle interventions: Identification of health behaviour patterns by cluster analysis in a German 50+ survey. Eur. J. Public Health.

[43] Fleary S.A., Nigg C.R. (2019). Trends in Health Behavior Patterns Among U.S. Adults, 2003–2015. Ann. Behav. Med..

[44] Sarhan I., Ehymmayed H.M., Mahmood S.E. (2023). Decision tree in obesity level classification of Northern Technical University students. NTU J. Agric. Vet. Sci..

[45] Iparraguirre-Villanueva O., Mirano-Portilla L., Gamarra-Mendoza M., Robles-Espiritu W. (2024). Predicting obesity in nutritional patients using decision tree modeling. Int. J. Adv. Comput. Sci. Appl..

[46] Trujillano J., Serviá L., Badia M., Serrano J.C.E., Bordejé-Laguna M.L., Lorencio C., Vaquerizo C., Flordelis-Lasierra J.L., Martínez de Lagrán I., Portugal-Rodríguez E. (2025). Methodological Review of Classification Trees for Risk Stratification: An Application Example in the Obesity Paradox. Nutrients.

[47] Salinas-Mandujano R.G., Reynoso-Camacho R., Salgado L.M., Ramos-Gomez M., Pérez-Ramírez I.F., Aguilar-Galarza A., Moreno-Celis U., Anaya-Loyola M.A. (2023). A New Approach Using BMI and FMI as Predictors of Cardio-Vascular Risk Factors among Mexican Young Adults. Eur. J. Investig. Health Psychol. Educ..

[48] Pourshahidi L.K., Wallace J.M., Mulhern M.S., Horigan G., Strain J.J., McSorley E.M., Magee P.J., Bonham M.P., Livingstone M.B. (2016). Indices of adiposity as predictors of cardiometabolic risk and inflammation in young adults. J. Hum. Nutr. Diet..

[49] Gutiérrez-Rojas C.A., Cruz-Soto R., Sánchez-Muñoz V., Romero A., Mosti-Molina M., Sánchez-Aguilar H.A., Velázquez-Fernández D., Herrera M.F. (2020). Does FMI Correlate Better than BMI with the Occurrence of Metabolic Changes in Obese Patients? Study Based on 2007 Consecutive Mexican Patients. Obes. Surg..

[50] Liu P., Ma F., Lou H., Liu Y. (2013). The utility of fat mass index vs. body mass index and percentage of body fat in the screening of metabolic syndrome. BMC Public Health.

