# Supplementary material for: Dose–Response Relationships Between Physical Activity, Dietary Behaviors, and Excess Body Weight: Identification of Behavioral Risk Patterns
Source: Nutrients. 2026 Jun 28;18(13):2104. doi: 10.3390/nu18132104 (PMC13362979; doi:10.3390/nu18132104)
Supplement: Supplementary file 1 [file nutrients-18-02104-s001.zip › nutrients-4360256-supplementary.pdf]

Supplementary Table S1. Behavioral threshold ranges for BMI- and FMI-defined risk zones across physical activity levels.

model	outcome	PA_level	risk_zone	min_healthy_index	max_healthy_index	min_unhealthy_index	max_unhealthy_index	mean_predicted_risk
BMI model	BMI-defined overweight/obesity	Low PA (-1 SD)	Elevated risk (20-30%)	-2.5	1.22	-1.19	2.5	0.244
BMI model	BMI-defined overweight/obesity	Low PA (-1 SD)	High risk (>=30%)	-2.5	-0.29	0.32	2.5	0.360
BMI model	BMI-defined overweight/obesity	Low PA (-1 SD)	Low risk (<10%)	-1.53	2.5	-2.5	1.46	0.067
BMI model	BMI-defined overweight/obesity	Low PA (-1 SD)	Moderate risk (10-20%)	-2.5	2.5	-2.5	2.5	0.144
BMI model	BMI-defined overweight/obesity	Average PA	Elevated risk (20-30%)	-2.5	1.69	0.18	2.5	0.243
BMI model	BMI-defined overweight/obesity	Average PA	High risk (>=30%)	-2.5	-0.15	1.19	2.5	0.354
BMI model	BMI-defined overweight/obesity	Average PA	Low risk (<10%)	-2.5	2.5	-2.5	1.39	0.055
BMI model	BMI-defined overweight/obesity	Average PA	Moderate risk (10-20%)	-2.5	2.5	-1.33	2.5	0.144
BMI model	BMI-defined overweight/obesity	High PA (+1 SD)	Elevated risk (20-30%)	-2.5	2.33	0.89	2.5	0.243
BMI model	BMI-defined overweight/obesity	High PA (+1 SD)	High risk (>=30%)	-2.5	0.08	1.63	2.5	0.347
BMI model	BMI-defined overweight/obesity	High PA (+1 SD)	Low risk (<10%)	-2.5	2.5	-2.5	1.39	0.042
BMI model	BMI-defined overweight/obesity	High PA (+1 SD)	Moderate risk (10-20%)	-2.5	2.5	-0.29	2.5	0.145
FMI model	FMI-defined excess fat	Low PA (-1 SD)	Elevated risk (20-30%)	-2.5	-1.73	2.33	2.5	0.206
FMI model	FMI-defined excess fat	Low PA (-1 SD)	Low risk (<10%)	-2.5	2.5	-2.5	1.93	0.042
FMI model	FMI-defined excess fat	Low PA (-1 SD)	Moderate risk (10-20%)	-2.5	2.5	0.89	2.5	0.135
FMI model	FMI-defined excess fat	Average PA	Low risk (<10%)	-2.5	2.5	-2.5	2.03	0.035
FMI model	FMI-defined excess fat	Average PA	Moderate risk (10-20%)	-2.5	2.5	1.46	2.5	0.129
FMI model	FMI-defined excess fat	High PA (+1 SD)	Elevated risk (20-30%)	1.39	2.5	2.1	2.5	0.219
FMI model	FMI-defined excess fat	High PA (+1 SD)	Low risk (<10%)	-2.5	2.5	-2.5	2.5	0.028
FMI model	FMI-defined excess fat	High PA (+1 SD)	Moderate risk (10-20%)	-1.46	2.5	1.09	2.5	0.136
